# Percutaneous spinal endoscopy with unilateral interlaminar approach to perform bilateral decompression for central lumbar spinal stenosis: radiographic and clinical assessment

**DOI:** 10.1186/s12891-021-04100-3

**Published:** 2021-03-01

**Authors:** Jingbo Xue, Haoxiang Chen, Bin Zhu, Xuelin Li, Zhihua Ouyang, Shan Li, Zhun Xu, Yong Xie, Yiguo Yan

**Affiliations:** Department of Spine Surgery, The First Affiliated Hospital of University of South China, Hengyang, People’s Republic of China

**Keywords:** Endoscopic, Radiographic and clinical assessment, Central lumbar spinal stenosis, Magnetic resonance imaging, Spine stability

## Abstract

**Background:**

Recently, a percutaneous spinal endoscopy unilateral posterior interlaminar approach to perform bilateral decompression has been proposed for use in treatment of lumbar spinal stenosis, As a development and supplement to traditional surgery, its advantages regarding therapeutic effects and prognosis, such as minor soft tissue damage, little intraoperative blood loss, and a quick return to daily life. However, there are few analyses of this surgery with a follow-up of more than 1 year,we conducted this study in order to quantitatively investigate radiographic and clinical efficacies of this surgery for central lumbar spinal stenosis.

**Materials and methods:**

Forty-six patients with central lumbar spinal stenosis were enrolled from January 2017 to July 2018. The visual analog scale (VAS) for back pain and leg pain, Oswestry disability index (ODI), modified MacNab criteria were used to evaluate clinical efficiency at preoperative and postoperative time points. The intervertebral height index (IHI), cross-sectional area of the spinal canal (CSAC), calibrated disc signal (CDS) and spinal stability were examined to assess radiographic decompression efficiency via magnetic resonance imaging and X-ray at preoperative and postoperative time points.

**Results:**

The VAS score for lower back pain and leg pain improved from 7.50 ± 0.78 to 1.70 ± 0.66 and from 7.30 ± 0.79 to 1.74 ± 0.68, respectively, and the ODI improved from 72.35 ± 8.15 to 16.15 ± 4.51. In terms of modified MacNab criteria, 91.3% of the patients achieved good or excellent outcomes. Furthermore, significant changes after surgery were observed for the percentage of CSAC, increasing from 125.3 ± 53.9 to 201.4 ± 78 mm^2^; however, no significant differences were observed for the remaining measurement indicators.

**Conclusions:**

The clinical and radiographic efficacies of this surgery for central lumbar spinal stenosis were good in short-term follow-up, and this surgery did not cause meaningful changes in IHI, CDS, and spine stability in short-term follow-up. The effect of long-term follow-up needs further investigation.

## Introduction

Lumbar spinal stenosis (LSS) is a common disease that causes radiculopathy and back pain. It occurs when the neural structure is encroached on by the surrounding soft tissues and bones [1–3]. Traditional open paraspinal decompression is the standard surgical treatment for different types of lumbar spinal stenoses [4]. However, complications of open paraspinal decompression are common, including postoperative infection, non-fusion of the bone graft area, loosening and fracture of the internal fixation, persistent numbness and adjacent spondylosis [5–7] .Moreover, excessive removal of the paraspinal muscle may lead to paraspinal muscle denervation as well as adjacent segmental degeneration [8]. To reduce tissue damage, a percutaneous spinal endoscopy unilateral posterior interlaminar approach to perform bilateral decompression has been improved for LSS because of its advantages regarding therapeutic effects and prognosis, such as minor soft tissue damage, little intraoperative blood loss, short hospital stays, and a quick return to daily life and work [9–11]. However, there are few analyses of this surgery with a follow-up of more than 1 year involving radiographic assessments and clinical decompression data. Therefore, the objective of this study was to evaluate the efficacy of this surgery for central lumbar spinal stenosis radiographically and to examine clinical outcomes.

## Materials and methods

### Study population

This retrospective study included 52 patients who underwent our percutaneous spinal endoscopic decompression technique from January 2017 to July 2018; the postoperative follow-up averaged 21.3 ± 6.0 months. The patients were evaluated retrospectively with regard to clinical and radiologic outcomes as well as morphometric changes in the CSAC, CDS, IHI, and spinal stability (segmental angulation and segmental range of movement ROM). Six patients (13.0%) were lost to follow-up. Therefore, clinical and radiographic data were collected from the remaining 46 patients. The inclusion criteria for the study were as follows: 1) concordant imaging evidence of central lumbar spinal stenosis; 2) conservative measures for a minimum of 8 weeks with no alleviation of symptoms; 3) typical symptoms including intermittent claudication, severe neurological symptoms of the lower extremities, and waist and leg pain; The exclusion criteria were as follows: 1) previous spinal surgery, congenital dysplasia of the spine, fractures, infection or inflammation, or spinal tumor; 2) definitive segmental instability on preoperative dynamic radiographs or multiple segments of lumbar vertebrae requiring surgery. The study was approved by the Institutional Review Board at our hospital, and all patients gave informed consents.

### Surgical technique

The patient was placed in the prone position and given continuous epidural anesthesia. The operating table was adjusted to make the lumbar spine adapt to anteflexion. The pertinent vertebral lamina interspace was preliminarily located and marked according to X-ray images and landmarks of the lumbar vertebra. After routine disinfection of the surgical field and spreading of a sterile sheet, the puncture needle was placed at the lesion side on the marked vertebral plate interspace, and the location point was identified at the lower margin midpoint of the vertebral lamina. After positioning, a longitudinal incision of approximately 7 mm long was made through the skin, subcutaneous tissue, and deep facia at 0.5 cm–1 cm from the spinous process, with a 3-stage cannula used to expand the incision step by step. After the cannula position was satisfactory, the working cannula was inserted. A spinal endoscope was connected and inserted. The fibrofatty tissue on the surface of the ligamenta flava was cleaned with blue pincers via radiofrequency under endoscopy, revealing the ligamenta flava, articular process and vertebral lamina. The areas from the lower margin of the vertebral lamina to the attachment point on the ligamenta flava and the partial hyperplastic bone on the root of the spinous process were polished with a high-speed abrasive drill. A nerve hook was used to separate the ligamenta flava and dural sac to prevent adhesion, and the hypertrophic ligamenta flava was removed with blue pincers and bayonet forceps, exposing the inferior articular process on the operative side. Part of the inferior articular process was removed by using a high-speed abrasive drill to expose the superior articular process on the operative side and the articular surface of the superior and inferior articular processes. Part of the superior articular process was then removed by using the high-speed abrasive drill and bayonet forceps. Release of the spinal cord and nerve roots on the operative side after sufficient decompression was revealed by this probing. The endoscope was removed appropriately, and the working sleeve was placed under the spinous process to expose the contralateral vertebral lamina and articular processes. The protective sleeve of the high-speed abrasive drill was placed on one side of the dural sac and the contralateral nerve roots, and the lower margin of the contralateral vertebral plate vertebral lamina and the inferior articular process were polished to expose the contralateral superior articular process, followed by the contralateral superior articular process to expose and release the contralateral nerve roots. After release of the spinal cord and bilateral nerve roots, the bleeding was stopped completely using the tip of a radiofrequency probe. The incision was sutured with a single needle and then dressed with sterile patches.

### Outcome measurements

#### Clinical measurements

Clinical outcomes were assessed by 2 experienced clinical researchers using the VAS for back pain and leg pain preoperatively, postoperatively (the first day after operation), and at the last follow-up (more than 1 year after the operation). The ODI and the modified MacNab criteria were evaluated preoperatively and at the last follow-up. In addition, perioperative data and complications were recorded.

#### Radiologic measurements

Radiographs were blindly analyzed by two radiologists, and the mean of the measurements was calculated preoperatively, postoperatively, and at the last follow-up. Assessments were performed for the IHI, CSAC, CDS and spinal stability.

1. According to the research results of Koji Akeda et al. [12], disc height is expressed as the disc height index (DHI), which was calculated as [(Ha + Hp)/(Ds + Di)] × 100, where disc height measurements based on X ray were as follows: Ha, anterior disc height, Hp, posterior disc height, Ds, superior disc depth, Di, inferior disc depth.

2. The CSAC was measured from the narrowest section by using an imaginary line encircling the area between the facet and lamina.

3. The American Academy of Orthopedic Surgeons defines lumbar instability as an abnormal response to stress, as characterized by abnormal lumbar motion beyond the normal limit [13]. Stability was evaluated by flexion-extension radiographs to measure the segmental range of movement (ROM) and segmental angulation. Instability was defined as slippage of more than 4 mm or an intervertebral angle > 11° in flexion/extension radiographs.

4. The average standard intervertebral disc signal (CDS) proposed by Gay [14] was used: on the central cross-sectional T2WI of the intervertebral disc, the gray value of the central circular area of the L2 and L3 intervertebral disc was selected as the reference, and the gray value of the central area of the intervertebral disc of the surgical segment was compared with it. This ratio eliminates the influence of other parameters of MRI equipment and can reflect the signal strength of the intervertebral disc.

**Fig. 1:**
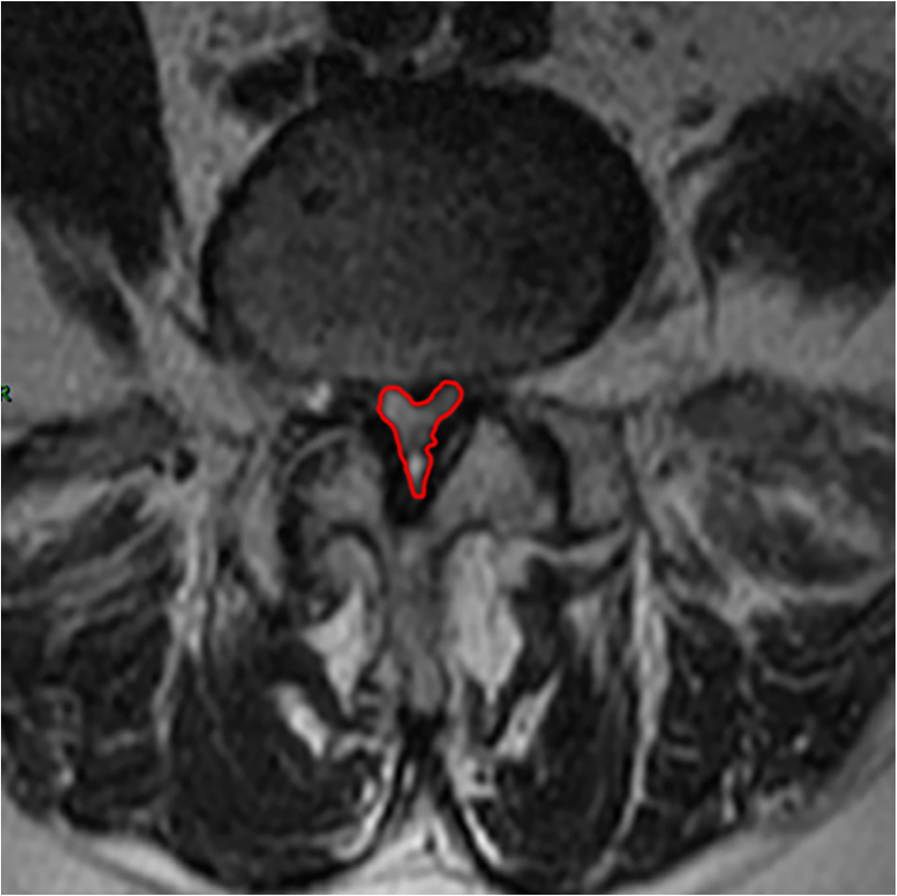
CSAC Before surgery

**Fig. 2:**
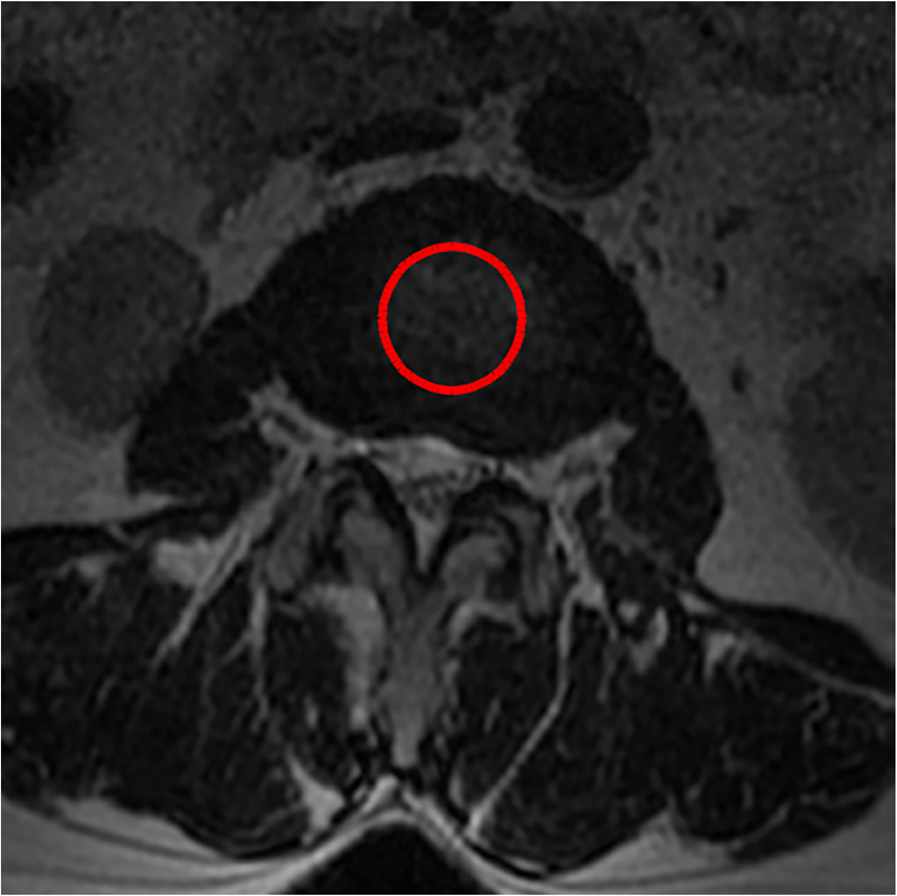
CDS Before surgery(L2,3)

**Fig. 3:**
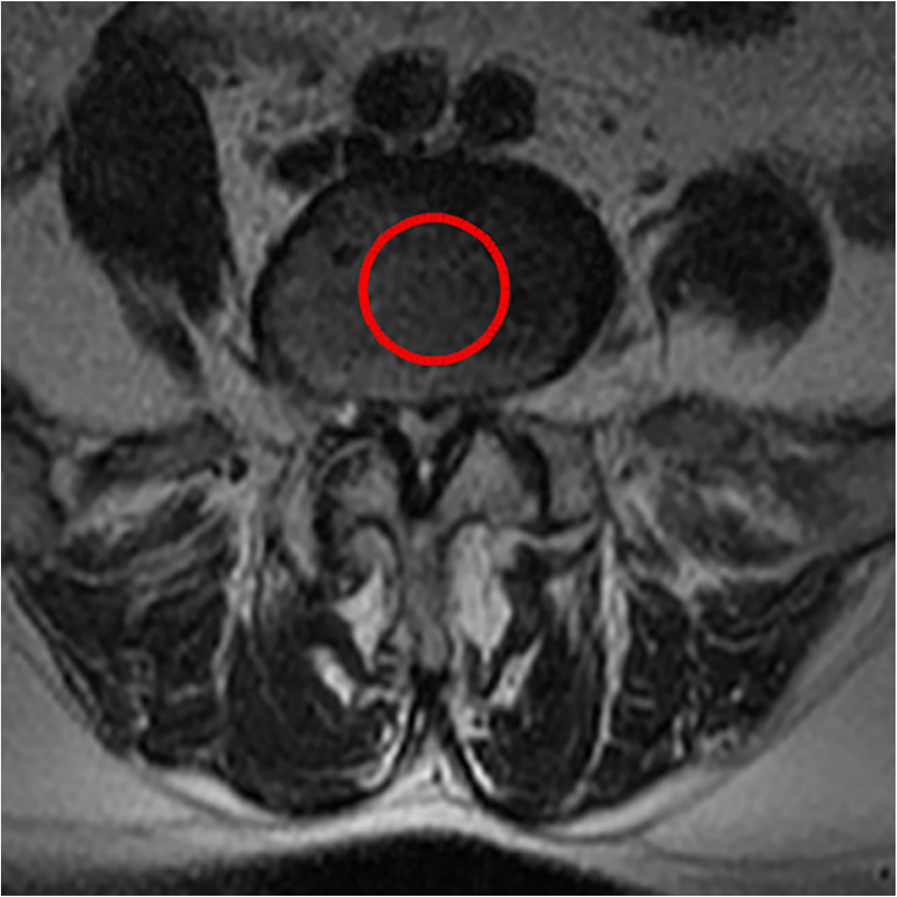
CDS Before surgery(L4,5)

**Fig. 4:**
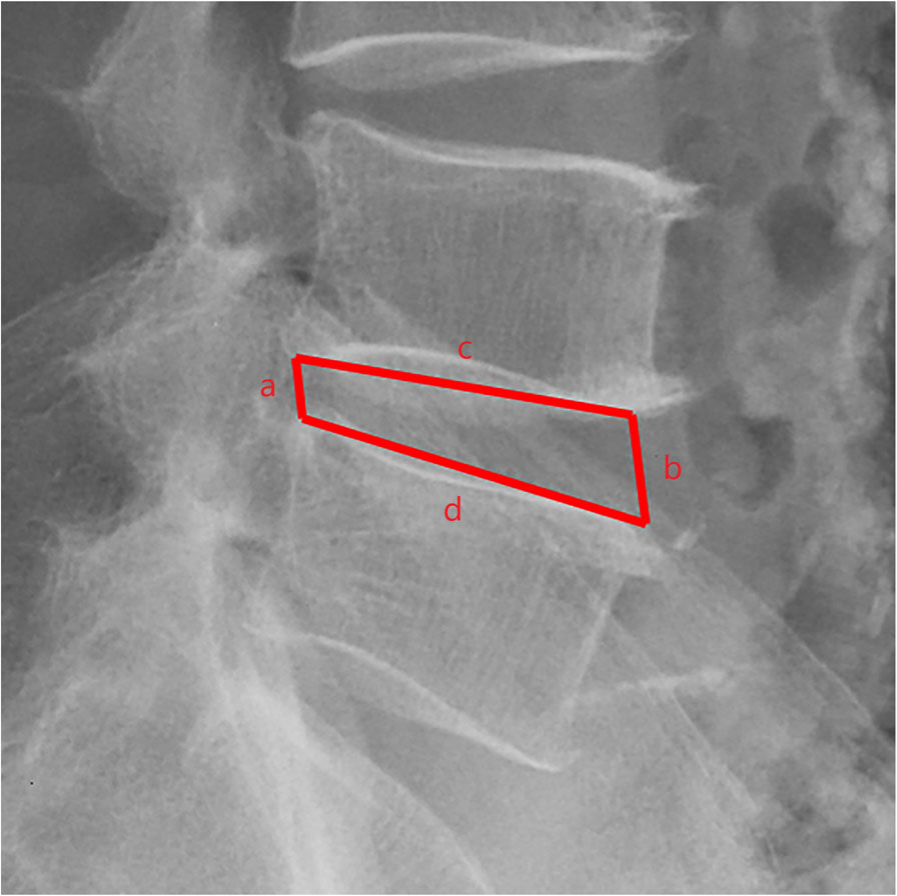
IHI Before surgery

**Fig. 5:**
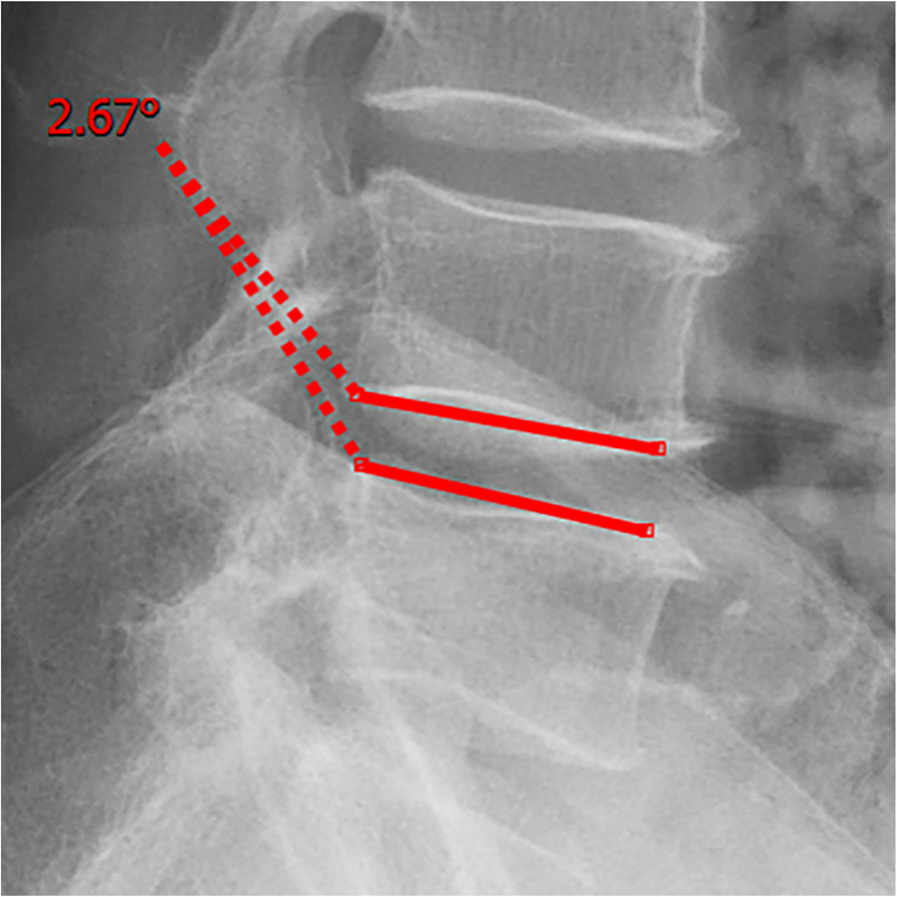
Segmental angulation Before surgery (flexion)

**Fig. 6:**
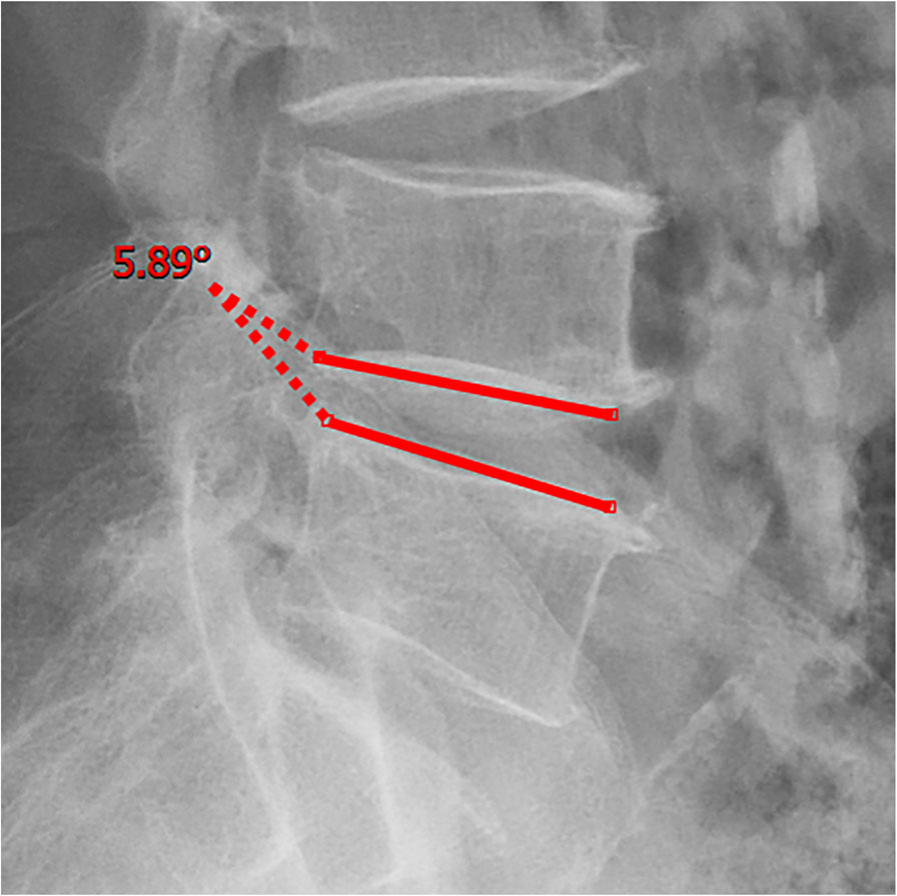
Segmental angulation Before surgery (extension)

**Fig. 7:**
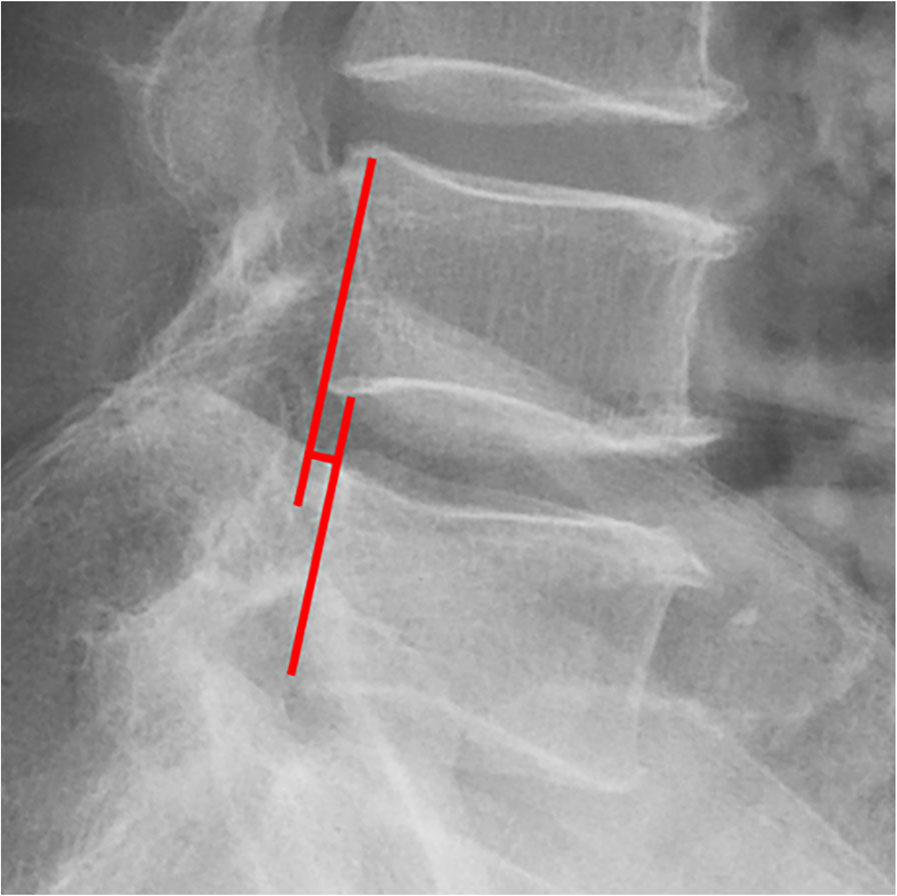
ROM Before surgery (flexion)

**Fig. 8:**
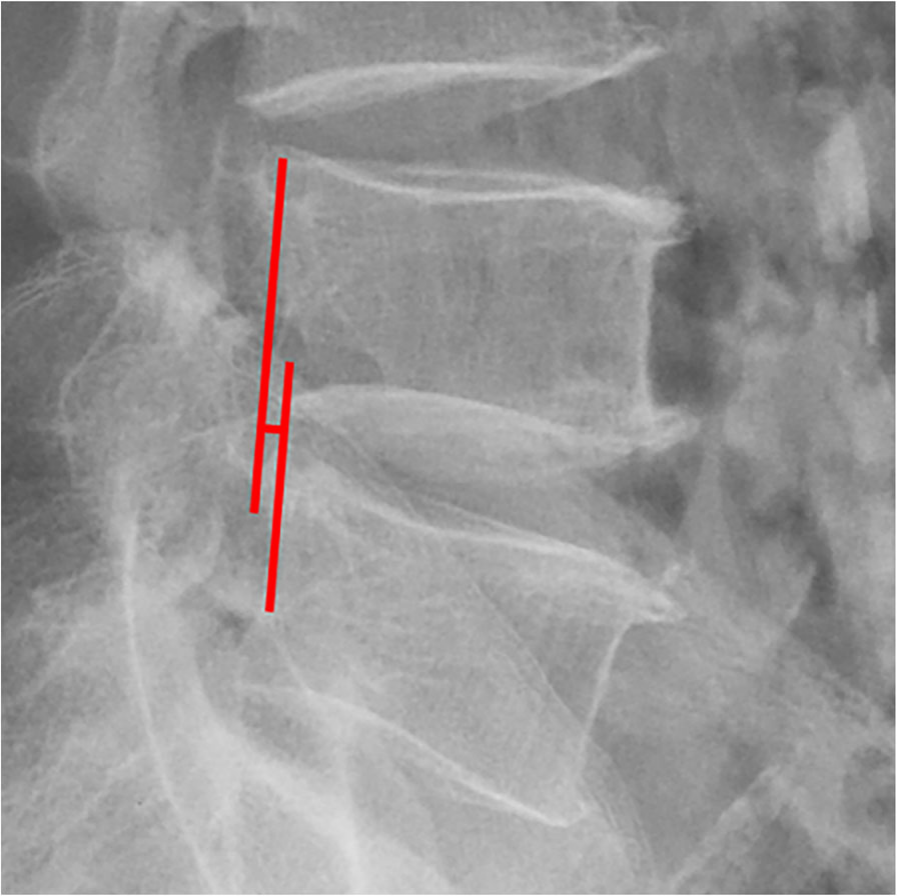
ROM Before surgery (extension)

**Fig. 9:**
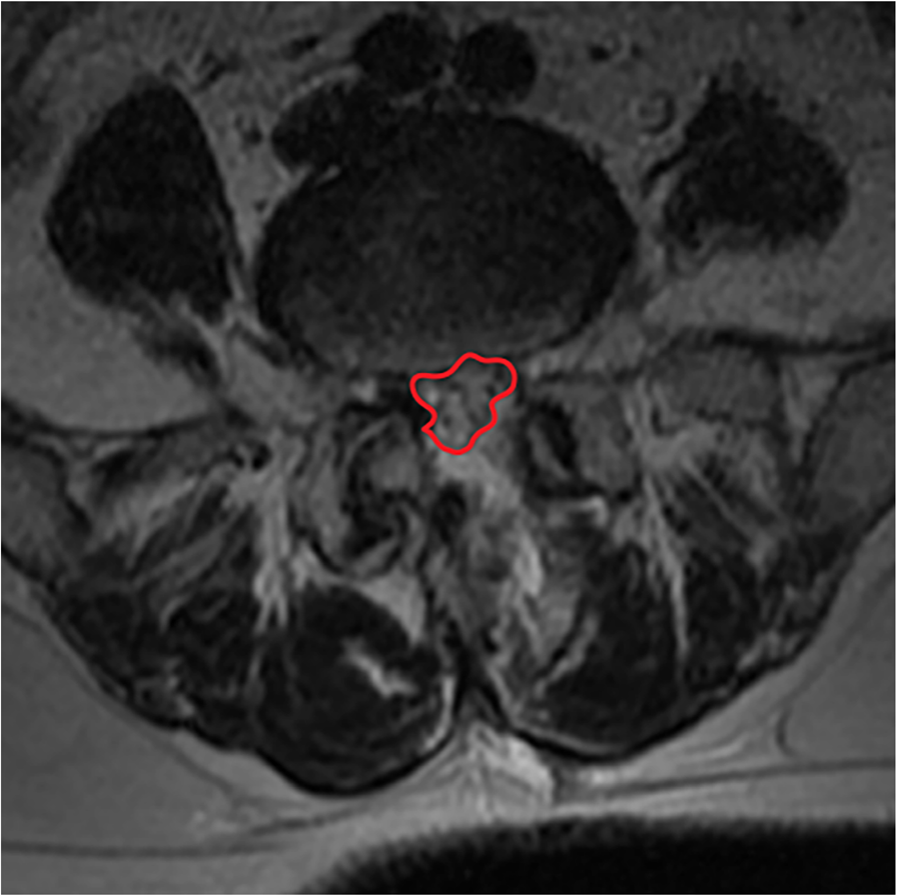
CSAC After surgery

**Fig. 10:**
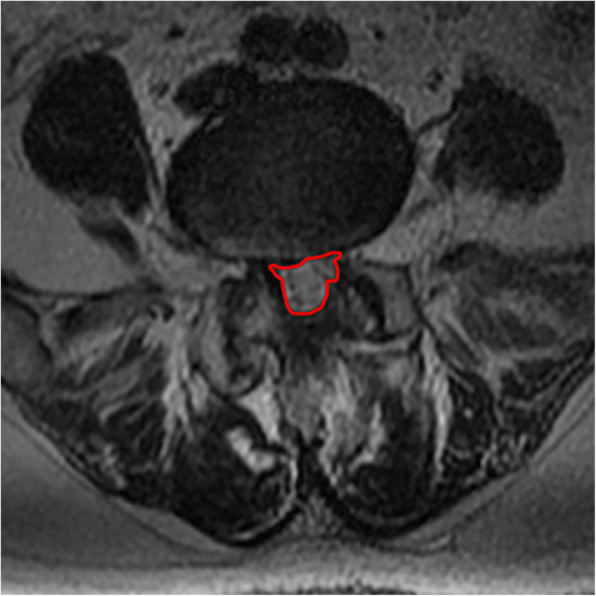
CSAC at the last follow up

**Fig. 11:**
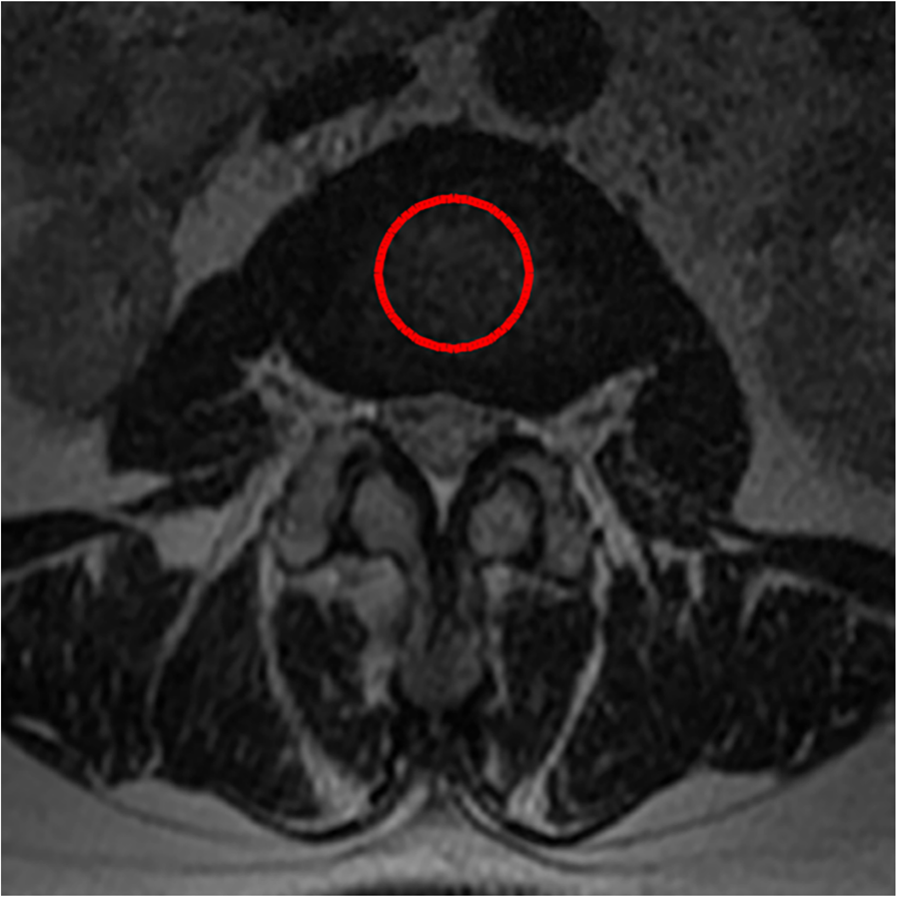
CDS at the last follow up(L2,3)

**Fig. 12:**
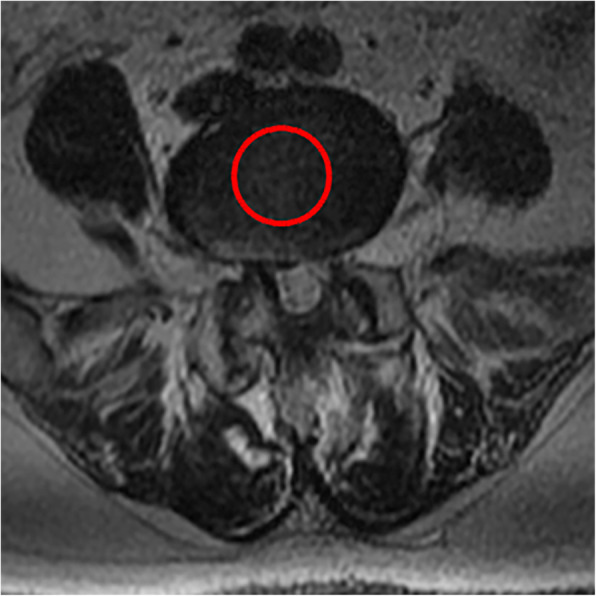
CDS at the last follow up(L4,5)

**Fig. 13:**
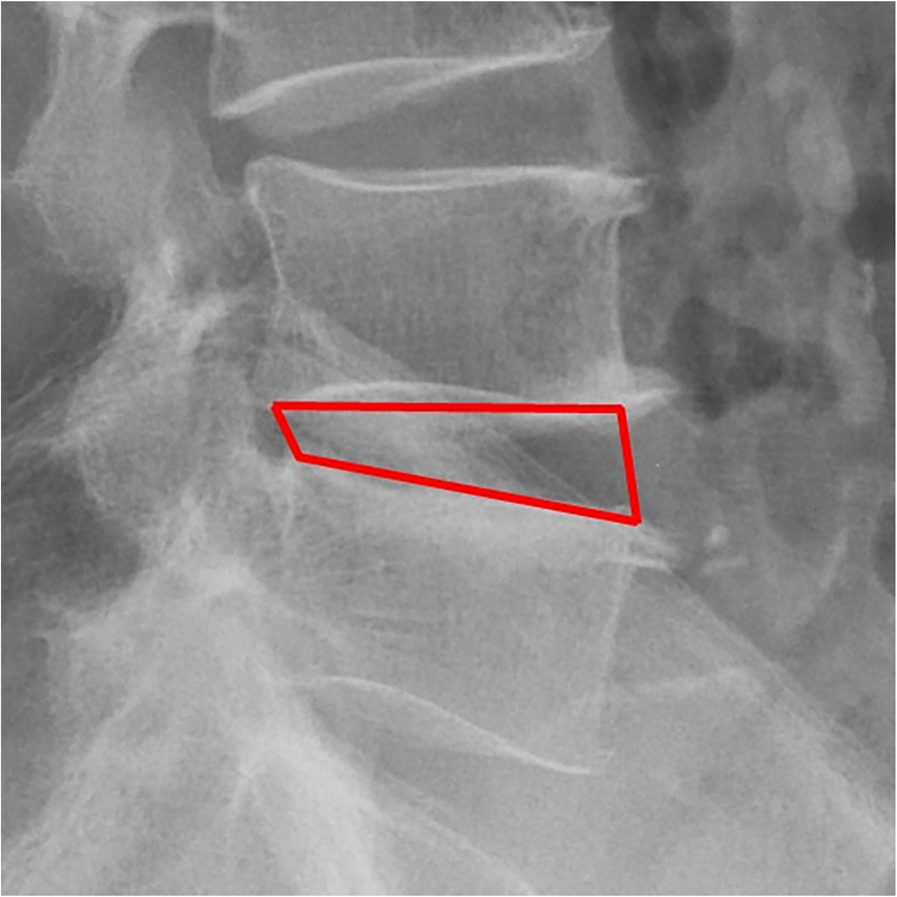
IHI at the last follow up

**Fig. 14:**
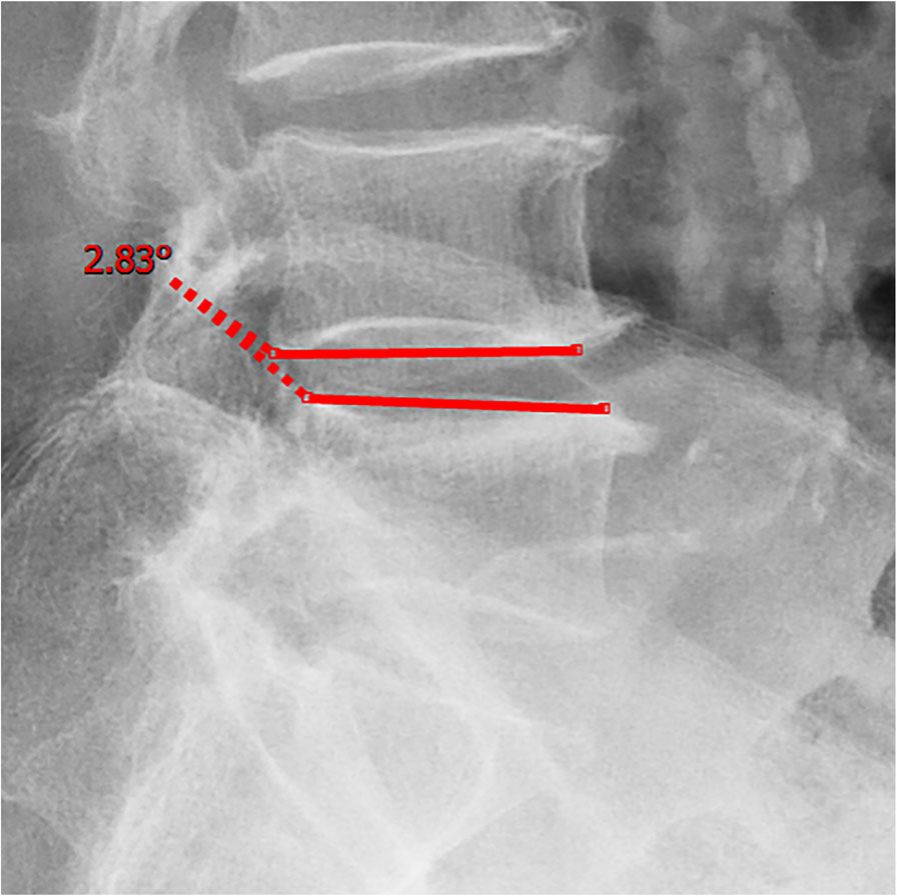
Segmental angulation at last follow up (flexion)

**Fig. 15:**
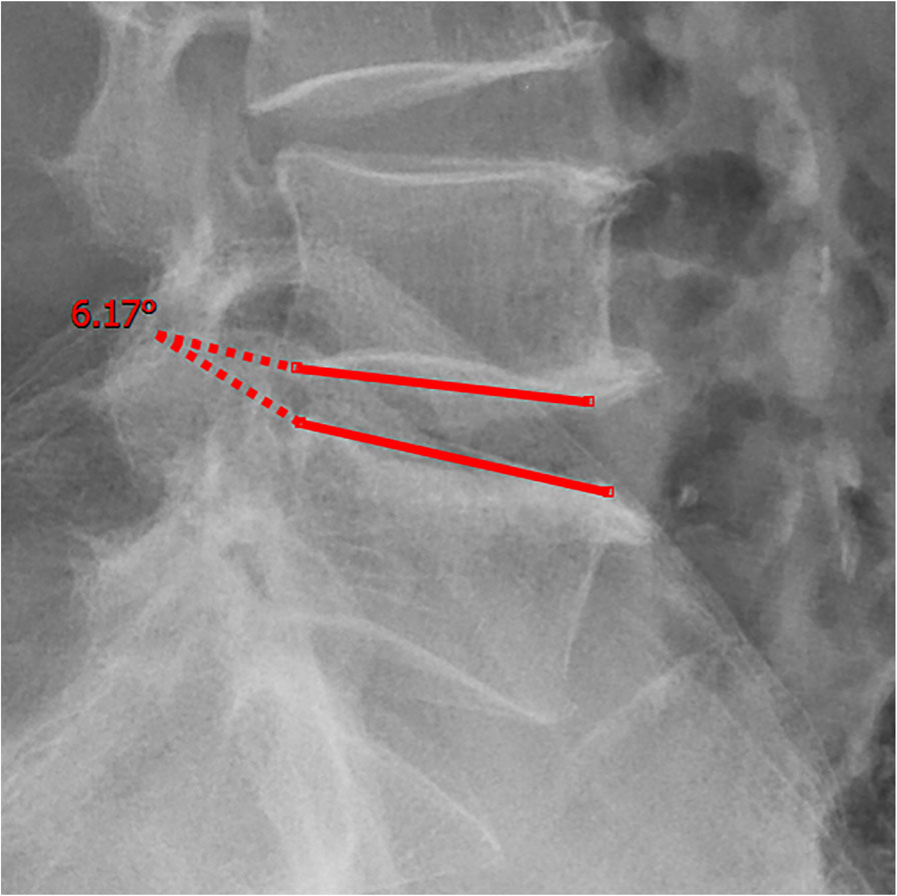
Segmental angulation at last follow up (extension)

**Fig. 16:**
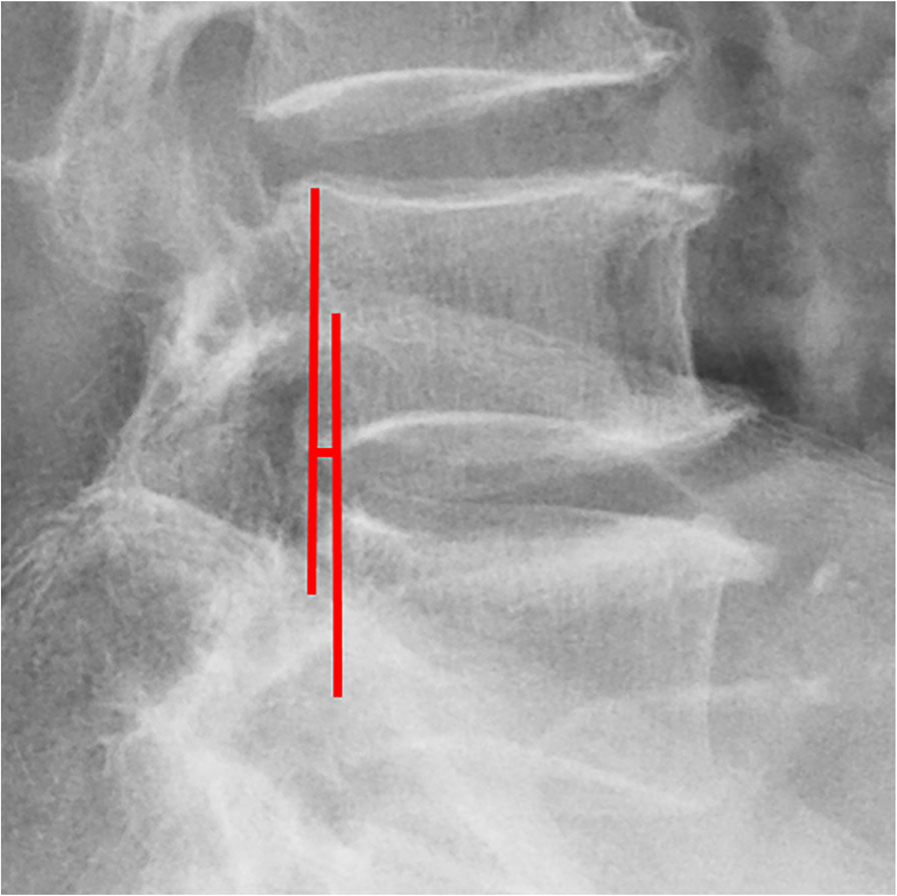
ROM at last follow up (flexion)

**Fig. 17:**
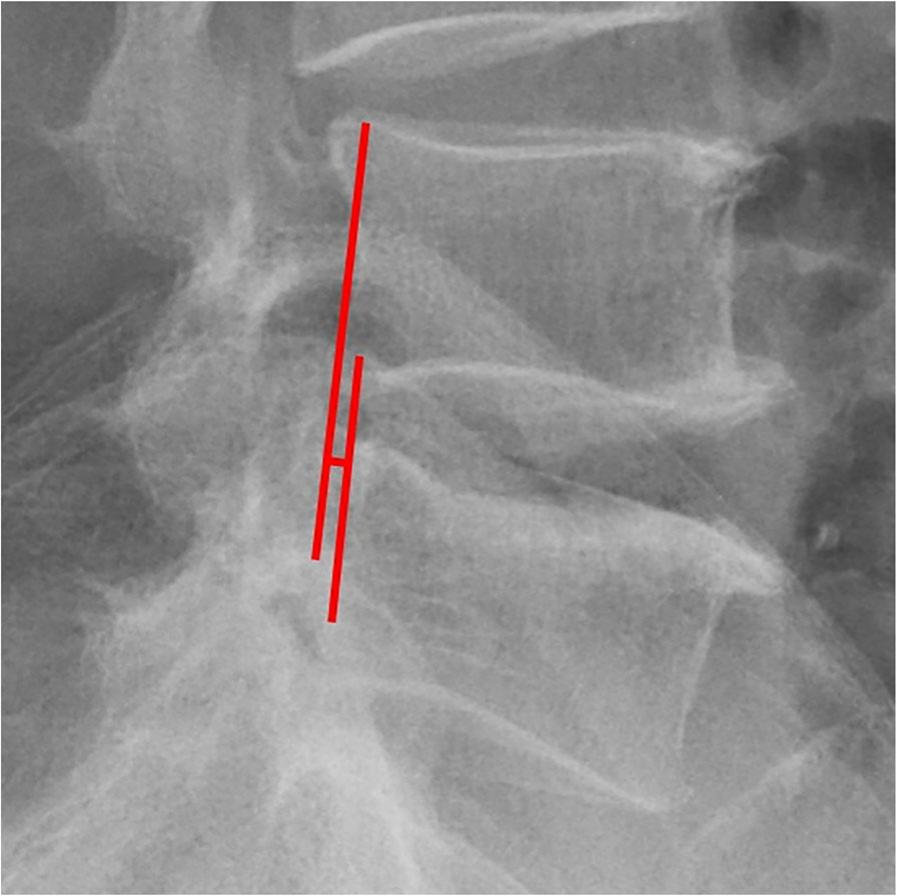
ROM at last follow up (extension)

All pre- and postoperative MRI scans and X-rays were obtained using the same machine, when possible, with the same settings (MRI:GE signa 1.0 T MRI; X-ray: DRX-Ascend Carestream). Postoperative MRI was performed within 48 h for all patients. MRI measurements were obtained using T1- and T2-weighted turbo spin echo sagittal images (TR/TE, 3200/102 for T1-weighted images and 3900/110 for T2-weighted images; slice thickness, 5 mm; slice gap, 1 mm; matrix, 512 × 256; field of view, 20 cm). The dimensions of all parameters were automatically calculated using available software (Carestream Vue PACS, Canada). The measurement method is shown in Figs. 1, 2, 3, 4, 5, 6, 7, 8, 9, 10, 11, 12, 13, 14, 15, 16 and 17

### Statistical analysis

Statistical analyses were performed using SPSS, Version 24.0 (SPSS, Chicago, Illinois, USA), and the level of significance was defined as *P* < 0.05. The repeated measures method was performed for variance analysis to compare clinical and radiographic data pre- and postoperatively and at the last follow-up.

## Results

### Demographics

The study participants included 25 women and 21 men, with a mean age (years) of 55.8 years (range, 21–82 years). A total of 46 vertebral levels were decompressed in the 46 patients: 19 (41.3%) underwent decompression at L5-S1, 23 (50.0%) at L4-L5, and 4 (8.7%) at L3-L4. Two patients experienced dural tears (no symptoms), In one case, we covered the tear of dural sac with gelatin sponge during operation, and in the other case, we sutured the tear of dural sac directly under endoscope. One patient had a transient decrease in muscle strength of the right lower limb (from grade V to grade III), which was characterized by a decrease in the strength of the extensor dorsi of the thumb. After conservative treatment (such as Methylprednisolone) involving relieving neuroedema, the muscle strength gradually recovered at 1 week after the operation. Another patient exhibited aggravation of pain in both lower limbs, The symptoms were relieved after hematoma puncture and drainage with 5 ml syringe connected with Arterial long needle (Type-B sonic can be used if necessary). At the end of the operation, we often stop saline perfusion to check for bleeding and usually do not place a drainage tube to reduce the risk of infection. Oxygen therapy was given to the patient for 5–10 min before anesthesia to improve the patient’s tolerance to the operation, and appropriate sedation treatment was given to the over-stressed patient. It is necessary to observe the patient in the operating room for 15–30 min after the operation. Only if the patient’s vital signs are stable can he/she be sent to the hospital bed. The whole process mean time (including operation time) was 142 min (range, 95–175 min), and the average hospital stay was 6–14 days (average 8.6 days), excluding the time of waiting for MRI and completing routine examination before operation, the postoperative hospital stay of the patient was 2–5 days (average 3.5 days). The patient demographics are listed in Table 1.

**Table 1:** Patient demographics

Variable	No.
Patients (no.)	46
M:F	25:21
Mean age (year)	55.8 (21–82)
Operated levels (*n* = 46)	46
L3–4	4 (8.7%)
L4–5	23 (50.0%)
L5–S1	19 (41.3%)
Number of postoperative follow-ups (month)	19.9 ± 6.0
Mean operation time (min)	142 (95–175)
Mean hospital stay (days)	8.6 (6–14)
Complications	4 (8.7%)

### Clinical outcome

The VAS score for lower back pain improved from 7.50 ± 0.78 preoperatively to 3.46 ± 0.66 postoperatively and 1.70 ± 0.66 at the last follow-up. The VAS score for leg pain also improved from 7.30 ± 0.79 preoperatively to 3.80 ± 0.69 postoperatively and 1.74 ± 0.68 at the last follow-up. For ODI, improvement from 72.35 ± 8.15 preoperatively to 16.15 ± 4.51 at the last follow-up was observed. These results are summarized in Table 2. According to the modified MacNab criteria, the overall results were excellent in 22 cases (47.8%), good in 20 (43.5%), fair in 4 (8.7%), and poor in 0 (0%); excellent or good results were achieved in 91.3% of cases.

**Table 2:** Comparison of VAS score and ODI (^−^x ± s)

Time	Low back pain VAS score	Leg pain VAS score	ODI (%)
preoperative	7.50 ± 0.78	7.30 ± 0.79	72.35 ± 8.15
postoperative	3.46 ± 0.66	3.80 ± 0.69	–
last follow-up	1.70 ± 0.66	1.74 ± 0.68	16.15 ± 4.51

### Radiologic outcome

Postoperative MRI showed decompression of the pathologic segment in all patients.. The average value of the CSAC according to observers 1 and 2 increased significantly from 125.3 ± 53.9 mm^2^ preoperatively to 201.4 ± 78.0 mm^2^ postoperatively (*P* < 0.01) and to 202.2 ± 72.2 mm^2^ at the last follow-up (*P* < 0.01). The IHI (%) based on X-ray was 31.77 ± 5.39 preoperatively, and 30.98 ± 5.41 at the last follow-up (*P* > 0.05), with no significant differences. The CDS (%) was 93.95 ± 37.95 (preoperative) and 88.96 ± 29.05 (at the last follow-up) (*P* > 0.05). (Table 3). Additionally, the segmental angulation for lumbar extension at the last follow-up was 7.01° ± 1.74°, and that for lumbar flexion was 6.51° ± 1.56°. The ROM (extension) at the last follow-up was 2.87 ± 0.53 mm; the ROM (flexion) at the last follow-up was 2.78 ± 0.54 mm. (Table 4).

**Table 3:** Comparison of CSAC, IHI(%), CDS(%) (^−^x ± s)

Time	CSAC	IHI(%)	CDS(%)
preoperative	125.3 ± 53.9	31.77 ± 5.39	93.95 ± 37.95
postoperative	201.4 ± 78.0	–	–
last follow-up	202.2 ± 72.2	30.98 ± 5.41	88.96 ± 29.05

**Table 4:** Comparison of spine stability (^−^x ± s)

Time	segmental angulation (flexion)	segmental angulation (extension)	The ROM (flexion)	The ROM (extension)
preoperative	6.21 ± 2.16	7.27 ± 1.75	2.73 ± 0.72	2.95 ± 0.64
last follow-up	6.51 ± 1.56	7.01 ± 1.74	2.78 ± 0.54	2.87 ± 0.53

## Discussion

### Radiographic assessment and quantitative analysis

#### Spinal degeneration

Eun et al. [15] reported an average disc height reduction of 18.8% at 11 years after endoscopic discectomy. Our surgical technique was able to obtain a visual angle of the dorsal structure of nerve tissue, for which it can safely be managed under the monitoring of microscopic visual field, including hypertrophic ligament tissue and the cohesive hyperplastic articular process. If necessary, the nerve structure can even be pushed medially to complete the treatment of its lateral and ventral compression [16]. In our series, we removed the hypertrophic ligamentum flavum and some lumbar facet joints during the operation, Lumbar facet joints play an important role in regulating the range of motion of the lumbar spine, carrying and transmitting the axial compression load of the spine, and maintaining the mechanical stability of the spine [17–19]. Biomechanical testing of isolated spinal segments has demonstrated that up to 33% of the total axial load of the spine segment can be borne by facet joints [20, 21]. Mechanical stress controls intervertebral disc matrix metabolism by affecting the biological behavior of intervertebral disc cells [22, 23] and mechanical stress plays an important role in the progression of intervertebral disc degeneration .so we want to know whether the change of axial load of the spine caused by partial resection of the facet joint in our endoscopic surgery will affect the intervertebral disc, so we detected the changes of IHI and CDS of the intervertebral disc. Fortunately, the postoperative IHI was basically unchanged from its preoperative value: the IHI had decreased slightly (no statistical significance) at more than 1 year after the operation. However, we do not consider that the slight decrease in disc height was due to degenerative processes and invasiveness of the discectomy [24, 25], as the central spinal canal stenosis in most of the patients was caused by hypertrophy of the ligamentum flavum or joint facet osteophytes in the spinal canal. And we just simply removed the hypertrophic ligamentum flavum or proliferative osteophyte rather than the normal intervertebral disc. Overall, our surgical technique is not suitable for patients with intervertebral foramen compression or stenosis, and we prefer to use a transforaminal approach for patients with intervertebral foramen stenosis with unilateral symptoms. The water content of the normal intervertebral discs was high, and the signal was evenly distributed on T2WI. When the synthesis and decomposition of the extracellular matrix become unbalanced due to a variety of factors [26], changes in the biochemical components of the nucleus pulposus, dehydration of intervertebral disc tissue and proteoglycan decomposition cause intervertebral disc degeneration. The degree of lumbar intervertebral disc degeneration was evaluated according to the signal intensity of the intervertebral disc on T2WI. In addition, Imai Y et al. [27] showed that the frequency and intensity of biomechanical load has an important influence on the degeneration of the lumbar intervertebral discs. In the present study, there were no significant changes in the CDS on follow-up compared to before the surgery. Combined with the analysis of IHI and lumbar stability, the stability of the lumbar spine was not damaged, and There is no significant change in the strength, direction and frequency of biomechanical loads.

Thus, we believe that this surgery will not accelerate the degeneration of the intervertebral disc in the short term, though the medium- and long-term effects on the intervertebral discs need further follow-up.

#### Spinal stability

Postoperative instability is a major concern in decompression surgery. In general, excessive removal of the facet joints has been associated with destabilization of the spine [28, 29]; traditional open paraspinal decompression peels off the paraspinal muscles and removes the lamina and spinous process extensively, and it is thus easy to damage the structure of the posterior column complex, resulting in iatrogenic segmental instability and spondylolisthesis [30, 31]. In our study, no postoperative lumbar instability occurred in the patients with stable lumbar spines after operation at the last follow-up, with no significantly increase in ROM and segmental angulation after the operation. Because the most of the posterior longitudinal ligament complex structures of the spine, such as the supraspinous ligament, interspinous ligament, facet joint and contralateral bony lamina, were protected, minimizing the risk of iatrogenic segmental instability. As Hamasaki et al. [32] confirmed using biomechanical evaluation in cadaver lumbar specimens, a unilateral approach for bilateral decompression with the preservation of posterior elements prevents up to 80% of the native anatomic stiffness. These findings are also supported by a finite element study conducted by Bresnahan et al. [33], and other articles report similar results. For example, Sasai et al.’s study [34] demonstrated no statistically significant increases in dynamic intervertebral angles and dynamic slip in patients undergoing microscopic bilateral decompression via the unilateral approach. According to Postacchini F et al.’s study [35], there was an association between well-maintained disc height and postoperative spondylolisthesis. Nonetheless, as we discuss above, the IHI remained basically unchanged before and after operation. In other words, the stability of the segmental spine was not be damaged by this operation in the short term.

#### Lumbar spinal canal decompression

The CSAC values consistently increased after surgery. The mean CSAC significantly increased from125.3 ± 53.9 mm^2^ to 202.2 ± 72.2 mm^2^, which was the most notable increase among the measurements. Our study also found that clinical symptoms improved with an increase in the CSAC. The statistically significant improvements in morphometric measurements also supported the clinical outcomes, and improvements in the mean CSAC indicate adequate central decompression of the stenotic lesion. Some scholars believe that lumbar spinal stenosis begins at the intervertebral disc [36]. According to our analysis, pathological changes such as intervertebral disc degeneration and herniation, posterior marginal hyperosteogeny, posterior longitudinal ligament thickening, lamina thickening, ligamentum flavum hyperplasia and even ossification [37]. inferior articular process degeneration and hyperplasia can cause bony or nonbony stenosis of the lumbar spinal canal. The effects include compression of the dural sac and changes its shape from a regular round or oval edge to an irregular edge. The MRI scans showed a decrease in the cross-sectional area of the dural sac, and stenosis of the central spinal canal was demonstrated by changes in the relevant area and diameter based on imaging data [38]. Similarly, these pathological changes can cause clinical systems both directly by mechanical compression of the spinal cord in the spinal canal and due to the relative decrease of the activity space of the spinal cord in the spinal canal during spinal movement, causing mechanical stimulation and compression [39, 40] or affecting the blood flow of the spinal cord [41]. After the pathological tissue of the compressed dural sac is fully excised by total spinal endoscopy, the original shape of the dural sac can be restored under the action of intracapsular cerebrospinal fluid pressure, relieving compression of the epidural vessels. MRI scans in our study showed that the area of the central spinal canal after the operation was significantly larger than that before the operation. In addition, the clinical symptoms and VAS and ODI scores had well improved, which indicated that our operation for central lumbar spinal stenosis was efficiently achieved.

### Analysis of the clinical curative effect

In recent years, spinal endoscopy technology has developed rapidly. Compared with traditional open surgery, This spinal endoscopic has the following advantages. 1. The risk of anesthesia is low. Most anesthesia is continuous epidural anesthesia, with risks that are lower than general anesthesia, and patients can respond to intraoperative discomfort in a timely manner, thus playing a role similar to neuromonitoring. 2. There is minimal surgical trauma. Indeed, the microincision on the back is called a “keyhole”. The working channel rotates into the intermuscular space during the operation, minimizing the mechanical trauma behind the spine, which can effectively reduce the denervation of paraspinal muscles and reduce scar adhesion in the spinal canal 3. The procedure leaves the visual field clear, and safety during the operation is high. The endoscope has good lighting, a 25° expandable visual field, and a magnified imaging system [42]. Continuous water pressure irrigation is also helpful for reducing the risk of infection. 4. The therapeutic effect is good, In our study, a significant improvement in the ODI score was seen in patients undergoing this operation, with > 90% of them having good or excellent scores per the modified MacNab criteria, However, this operation also has some shortcomings. The main is that the operation requires high operation requirements of surgeons, the learning curve is steep [43], and beginners of this surgery are easy to cause some complications, like other spinal endoscopic techniques [44]. For example, when patients in the prone position, due to the long operation time and continuous irrigation of the spinal canal with Stroke-physiological saline solution, it may increases intracranial pressure and causes headache. Therefore, the complication can be effectively reduced by enlarging the bony spinal canal first and then resecting the ligamentum flavum during the operation. Therefore, it is necessary to pay attention to the contraindications of the operation: 1. For multi-segmental spinal canal stenosis, the operation is difficult with unsatisfactory decompression effect, and it is easy to cause complications such as dural sac tear, so this operation is not recommended. 2. For patients with severe spinal canal stenosis caused by hyperplastic osteophytes, there is little space for nerve activity in the spinal canal, and endoscopic decompression has the risk of aggravating nerve injury, so open surgery should be considered first .3. When there is lumbar instability, open surgery combined with internal fixation may be a safer approach. The patient’s age, body size and cognitive degree should be taken into consideration.

At the same time, we noticed that the big working channel endoscopes techniques such as iLESSYS® Delta system [45] are also appropriate surgical methods for the treatment of central lumbar spinal stenosis, which are equipped with a larger size working cannula and endoscopic instruments, and permits big osteophytes or soft tissues to be removed without extra maneuvers under good endoscopic visualization [46, 47], and the operation time is also shorter than our technology, but in order to broader endoscopic field of view, it also caused more damage to the tissue. However, The decompression of our spinal endoscopy is more accurate, and less damage to tissue, but the operation is difficult and the learning curve is steep, Comparing the two surgical techniques, we think that each operation has its own advantages, we can be more rational in the choice of surgical indications.

### Limitations

The current study has some limitations. First, the axial MRI sections in the follow-up MRI may not have been precisely the same sections or at the same angle as the first MRI. Additionally, the follow-up duration was relatively short for an evaluation of radiographic and clinical changes. Finally, no control group was used, which may have had some influence on the evaluation of the surgical effect.

## Conclusions

We aimed to describe this surgical technique and to demonstrate its radiographic efficacy by MRI as well as its clinical effect. Our outcome clearly showed that this operation resulted in sufficient decompression of the narrow spinal canal, We also found that this surgical technique will not damage the stability of lumbar surgical segments and will not accelerate degeneration of lumbar intervertebral discs in the short term. More than 1 year after the operation, the excellent and good rates of the clinical effect fully verified the beneficial, clinical effect of this operation. Our future work will focus on a randomized controlled study and long-term follow-up observations after this surgery.

## Data Availability

Data and materials used in this study can be made available upon reasonable request from Yiguo Yan.
